# A Review on Gut Remediation of Selected Environmental Contaminants: Possible Roles of Probiotics and Gut Microbiota

**DOI:** 10.3390/nu11010022

**Published:** 2018-12-21

**Authors:** Pengya Feng, Ze Ye, Apurva Kakade, Amanpreet Kaur Virk, Xiangkai Li, Pu Liu

**Affiliations:** 1 Gansu Key Laboratory of Biomonitoring and Bioremediation for Environmental Pollution, School of Life Science, Lanzhou University, Tianshuinanlu #222, Lanzhou 730000, Gansu, China; fengpy15@lzu.edu.cn (P.F.); yez16@lzu.edu.cn (Z.Y.); xkli@lzu.edu.cn (X.L.); 2 Ministry of Education Key Laboratory of Cell Activities and Stress Adaptations, School of Life Science, Lanzhou University, Tianshuinanlu #222, Lanzhou 730000, Gansu, China; apurva2017@lzu.edu.cn (A.K.); lzu_amanpreet@lzu.edu.cn (A.K.V.)

**Keywords:** environmental contaminants, remediation, probiotics, gut microbiota, foodborne

## Abstract

Various environmental contaminants including heavy metals, pesticides and antibiotics can contaminate food and water, leading to adverse effects on human health, such as inflammation, oxidative stress and intestinal disorder. Therefore, remediation of the toxicity of foodborne contaminants in human has become a primary concern. Some probiotic bacteria, mainly *Lactobacilli,* have received a great attention due to their ability to reduce the toxicity of several contaminants. For instance, *Lactobacilli* can reduce the accumulation and toxicity of selective heavy metals and pesticides in animal tissues by inhibiting intestinal absorption of contaminants and enhancing intestinal barrier function. Probiotics have also shown to decrease the risk of antibiotic-associated diarrhea possibly via competing and producing antagonistic compounds against pathogenic bacteria. Furthermore, probiotics can improve immune function by enhancing the gut microbiota mediated anti-inflammation. Thus, these probiotic bacteria are promising candidates for protecting body against foodborne contaminants-induced toxicity. Study on the mechanism of these beneficial bacterial strains during remediation processes and particularly their interaction with host gut microbiota is an active field of research. This review summarizes the current understanding of the remediation mechanisms of some probiotics and the combined effects of probiotics and gut microbiota on remediation of foodborne contaminants in vivo.

## 1. Introduction

The anthropogenic activities, rapid industrialization and urbanization have resulted in generation of hazardous toxic pollutants and consequent contamination of soil and water resources. For example, antibiotics (ABs) from medical waste, livestock manure and aquatic breeding have resulted in surface water contamination [1]. In addition, the large area of soil is contaminated by heavy metals (HMs) depositions and pesticides spraying [2]. It is reported that about 2.5 million hectares of soil area in Europe alone is a victim of pollution [3]. In China, mining has resulted in severe HMs contamination of 2.88 × 10^6^ ha of land, with an additional mean area of 46,700 ha polluted annually [4]. HMs and pesticides can accumulate in agricultural products grown in the contaminated soil [5,6]. Hence, these environmental contaminants are readily transmitted into human body through water and diet, exerting negative effects on human health, such as inflammation [7,8], oxidative stress [9,10,11] and intestinal disorder [12,13,14]. Remediation of contaminants originated from environment has thus become a primary concern worldwide. 

Many strategies have been developed for the remediation of soil and water contamination over the past decades, including physicochemical, microbial and phytoremediation methods. Phyto- and microbial remediation is regarded as useful approach with minimal site disruption [15], and eliminates the requirement for soil excavation and transport [16]. However, the total area that can be repaired in these traditional ways is far smaller than the total area of contamination. Thus, human exposure to contaminants is currently inevitable, and alternative methods are needed to protect not only the environment but also human against environmental contaminants.

Probiotics and live microbes that exert benefit on human health when supplemented in sufficient amounts [17], are considered as a promising tool for protection against foodborne contaminants. Evidences have shown that *Lactobacilli* can alleviate acute and chronic cadmium (Cd) toxicity [18,19], protect organisms against pesticides toxicity [20,21], reduce the risk of antibiotics associated diarrhea (AAD), and meantime rebalance the gut microbiota (GM) [22]. GM comprises about 3.8×10^13^ microorganisms inhabiting in the gastrointestinal tract (GIT), and the majority of these species belong to six bacterial phyla: *Firmicutes*, *Bacteroidetes*, *Actinobacteria*, *Proteobacteria*, *Fusobacteria*, and *Verrucomicrobia* [23,24]. GM has been known to play significant role in many physiological functions, such as regulating immunity [25] and metabolism [26], and also in the bioavailability and toxicity of various contaminants [27,28]. Mounting evidences have suggested that GM can be modified by probiotics and contribute to detoxication of environmental contaminants [29]. Nevertheless, the possible roles of GM and probiotics in mediating the remediation of foodborne contaminants in mammals have not received much attention, especially how the GM modified by probiotics interacts with contaminants and how these interactions are relevant to host health. The aim of this study was to summarize the impact of environmental contaminants including HMs, pesticides and ABs on GM and host physiology, with focus on the potential mechanisms of probiotics during bioremediation processes. It could help to gain a better comprehension of the remediation mechanism of probiotics and provide new perspectives for future applications with probiotics.

## 2. Effect of HMs, Pesticides and ABs on the Composition and Function of GM

HMs, pesticides and ABs currently used in diverse industrial and agricultural sectors and in daily life are leading to the spread of contaminants into the environment, therefore increasing health related problems worldwide. During the last few decades, as more roles of GM are revealed, investigators have paid more attention on the impact of contaminants on GM mostly by analyzing stool microbiome in rodents, poultry, and aquatics using high-throughput sequencing techniques. These studies have demonstrated that the GM imbalance is often correlated with the occurrence of disorders of energy metabolism, nutrient absorption, and immune system [30,31].

### 2.1. HMs 

HMs, such as Cd, chromium (Cr), arsenic (As), lead (Pb), nickel (Ni) are hard to be remediated and they exert high level of toxicity on animals and humans. About 40–60% of ingested metals are absorbed across the intestinal barrier [32,33], causing oxidative stress, inflammation, tissues damages, and gastrointestinal disorders [34].

HMs cause marked alterations in the composition of the GM (Table 1). First, a decrease in richness as well as the diversity of GM, is often observed after exposure to HMs [35,36]. Second, the ratio of *Bacteroidetes* to *Firmicutes* at phylum level is usually increased upon HMs exposure, which was thought to be associated with loss of body weight [37]. Recent studies revealed that exposure to Cd have contributed to profound effects on the microbiome in the intestinal tract of mice [38,39]. The ratio of *Firmicutes* to *Bacteroidetes* decreased significantly in mice treated with low (10 or 20 mg/kg) and high concentrations (100 mg/kg) of Cd [14,30]. Similar changes were also observed in mice exposed to As (10 mg/L) and Cr (VI) (100 mg/L) [29,40]. In Cr (VI)-treated mice, the proportion of *Bacteroidetes* and *Tenericutes* increased, and the proportion of *Firmicutes* declined, significantly. The exception to the tendency of alteration is Pb, where the ratio of *Bacteroidetes* and *Firmicutes* decreased [41]. Third, the influence of HMs on GM is usually dose-dependent. Higher concentration of Cd treatment posed a greater impact on intestinal flora than lower concentration [38].

The compositional and functional alterations of the GM are often linked to the intestinal and overall physical health of the host. Generally, the population of beneficial bacteria related to host physiology and biosynthesis was decreased and the number of pathogenic bacterial species correlated with the inflammation and oxidative stress was increased [29,42]. In Cd-treated mice, the abundance of beneficial bacteria such as *Bifidobacteri* and *Lactobacilli* was decreased significantly [38], whereas the relative abundance of harmful bacteria, *Clostridiales*, *Prevotella* and S24-7 was increased; and Cr (VI) induced the decrease of the relative abundance of *Lachnospiraceae* in mice [29]. *Prevotella* were associated with the transferable and colitogenic activity in a colitis mouse model [43,44]. *Clostridiales* affect the production of short chain fatty acids (SCFAs) [45,46]. SCFAs, mainly acetate, propionate and butyrate, are the ligands of G-protein-coupled receptor 41 (GPR41), GPR43, GRP109A, and also play important roles in the colonic epithelial cells maintenance, gluconeogenesis energy supply, and gut immunity [47,48]. *Lachnospiraceae* produce butyrate, with great impact in energy supply and the function of enterocytes [49]. Similarly in mice treated with Pb, the abundance of *Lactococcus, Enterorhabdus,* and *Caulobacterales* was decreased, and the abundance of *Desulfovibrionaceae, Barnesiella* and *Clostridium* was increased at the family level, which may be a contributing factor for the increase in weight and other diseases [41]. Other host phenotypes associated with the alteration of gut microbial communities include gut barrier impairment [50], increased oxidative stress in hepatocyte, hepatic inflammation and damage [29], rise in the levels of lipopolysaccharide in the serum, energy metabolism dysregulation [30], and even adult adiposity [51].

### 2.2. Pesticides 

Pesticides are widely applied in agriculture to resist insects, weeds, and plant pathogens to promote plant growth. The crops are exposed to pesticides, which can readily get into human GIT through daily diet. Low levels of pesticides exposure can give rise to long-lasting adverse effects on skin, endocrine, and especially nervous system by inducing generation of free radicals that might cause lipid peroxidation, DNA damage, cell death and possible carcinogenic effects [52,53,54].

Many researchers have demonstrated the essential role of GM in the metabolism of pesticides in host. Some pesticides are known to be metabolized by the enzymes produced by GM. Organophosphate insecticide chlorpyrifos get metabolized into a more toxic molecule 3,5,6-trichloro-2-pyridinol via biotransformation by GM, resulting in biologically relevant and toxic consequences on host health. Whereas certain bacterial species, e.g. *Pseudomonas* spp. (ATCC700113), *L. lactis*, *E. coli* and *L. fermentum* present in GIT, are capable of utilizing 3,5,6-trichloro-2-pyridinol as their sole carbon and energy source [55,56,57]. In turn, the composition and function of GM are profoundly affected by long-term exposure to pesticides, correlated with various metabolic and immune diseases [28].

Organophosphorus (OP) pesticide has been extensively applied since 1950s. Chlorpyrifos is a typical OP insecticide that can result in altered host metabolism, increased bacterial translocation, and alterations in GM compositions. For instance, chronic chlorpyrifos exposure in rats increased the abundance of opportunistic pathogens, and unfavorable metabolic-related strains, resulting in obese and diabetic phenotypes [58]. In addition, chlorpyrifos exposure affected the proliferation of subpopulations of some strains (*Enterococcus* spp., *Bacteroides* spp.) and increased bacterial translocation in spleen and liver of rats [59,60]. In the simulator of the human intestinal microbial ecosystem(SHIME) model, chlorpyrifos also had a great impact on the population of culturable bacteria, leading to an increase in *Enterobacteria*, *Bacteroides* spp., *Clostridia* count and decrease in *Bifidobacterial* count [60,61]. Similar experiments conducted in mice showed that the relative abundance of some key microbes was significantly altered under chlorpyrifos stress, with altered urine metabolites related to the metabolism of amino acids and energy, SCFAs, phenyl derivatives and bile acids [62]. Different bile acids can bind to different receptors and promote the absorption of dietary fats, regulate lipid and glucose metabolism, and shape the GM [63,64]. GM can transform bile acids and altered GM would influence the pool of bile acids and the host’s energy metabolism.

Malathion, diazinon and glyphosate are another three representatives of OP pesticide. In malathion-treated mice, gut microbiome development and quorum sensing were perturbed, with an increase in the relative abundance of bacterial genes associated to quorum sensing-related behaviors such as motility and pathogenicity [65]. Sex-specific impact on gut microbiome by diazinon was examined in a mouse model. Specifically, several bacterial genera, including *Bacteroidaceae_Bacteroides*, *Burkholderiales*_Other, *Clostridiaceae*_Other, and *Erysipelotrichaceae_Coprobacillus*, were only observed in male mice, while *Lachnospiraceae_Butyrivibrio Lachnospiraceae_Shuttleworthia*, and *Staphylococcaceae_Staphylococcus* were completely inhibited in males after diazinon exposure [66]. The effect of glyphosate on poultry microbiota was evidenced by the elevated resistance of pathogenic bacteria including *Salmonella entritidis*, *Salmonella gallinarum*, *Salmonella typhimurium*, *Clostridium perfringens* and *Clostridium botulinum*, and increased susceptibility of most of the beneficial bacteria such as *Enterococcus faecalis*, *Enterococcus faecium*, *Bacillus badius, Bifidobacterium adolescentis* and *Lactobacillus* spp. [67].

Organochloric pesticide (OCP), another type of common pesticide, interferes with intestinal flora, lipid metabolism, and tissue and body weight in animals. In mice, OCP induced increased abundance of *Firmicutes* and *Proteobacteria*, and decreased abundance of *Bacteroidetes*, *Verrucomicrobia*, and *Actinobacteria*. Meantime, the expression of genes involved in bile acid reabsorption by the terminal ileum was down-regulated, and compensatory expression of genes in synthesis of bile acids was up-regulated in the liver [68]. When permethrin was administered through diet in rat, it caused reduction in abundance of *Bacteroides-Prevotella-Porphyromonas* species and increase in the abundance of *Enterobacteriaceae* and *Lactobacillus* in fecal microbiota; altered SCFAs levels were registered over a 4-month period [69]. Pentachlorophenol exposure in gold fish led to an increased in the *Bacteroidetes* abundance and a decrease in the ratio of *Firmicutes* to *Bacteroidetes* in the gut, which played crucial roles in the reduction of body weight. *Bacteroides* genus within the *Bacteroidetes* phylum was significantly correlated with pentachlorophenol exposure dosage and duration [70].

Imidazole is widely used to inhibit fungus in agriculture. Recent studies revealed that GM dysbiosis induced by imidazole exposure is often associated with hepatic metabolism disorder and hepatic toxicity. When imazalil was orally given in zebrafish and mice, the abundance of *Bacteroidetes* was decreased, and *Firmicutes* increased in the gut at phylum level. In mice at the genus level, the abundance of *Lactobacillus* and *Bifidobacterium* decreased while those of *Deltaproteobacteria* and *Desulfovibrio* increased in response to imazalil exposure. In addition, the transcription of genes such as *Aco*, *Cpt1*, *Acc1*, *Srebp1a* and *Fas*, related to glycolysis and lipid metabolism was significantly decreased in the mouse liver [71,72]. In the mice that were exposed to carbendazim, the amounts of *Bacteroidetes* in the feces, and richness and diversity of GM in the cecum decreased significantly after the 5-day exposure. Analysis of operational taxonomic units (OTU) indicated that a total of 361 out of 3271 identified OTUs were significantly changed [31].

### 2.3. ABs 

Abs are widely used in stockbreeding, veterinary and human medicines [73,74]. Part of the ingested ABs by humans and animals can enter the environment through feces or urine [75]. Large quantity of ABs was detected in the ecosystem [76,77]. Hence, humans are readily exposed to antibiotic contamination passively in addition to medical route. The side effects of ABs range from relative mild ones, such as allergy, asthma, and diarrhea to severe ones, e.g., death [78].

ABs administration has been correlated with changes in the population structure of microbiome, which might be linked to a multitude of diseases. In particular, AAD and *Clostridium difficile* infections can be common following ABs treatment [79,80]. It has been previously shown that the microbial diversity was significantly reduced after treatment with ampicillin, streptomycin and clindamycin in the cecal and large intestine contents of mice. The *Bacteroidetes* population was drastically reduced, which never fully recovered following cessation of treatment, and the outgrowth of two dominant genus, *Stenotrophomonas* and *Xanthamonas* [81]. The predominant genus *Stenotrophomonas* is noteworthy since this highly antibiotic resistant bacterium is also a potential emerging opportunistic pathogen [82]. Treatment with clindamycin and ampicillin made the patients susceptible to *Clostridium difficile* infection and decreased *Clostridium scindens* count, which is a secondary modulator of bile acid metabolism [83]. A number of recent studies revealed that the abundance of *Proteobacteria* phylum in microbiota was significantly increased as a consequence of antibiotic administration [84,85,86]. *Proteobacteria* encompass a wide variety of pathogens, such as *Escherichia*, *Vibrio*, *Salmonella*, *Helicobacter*, *Yersinia*, *Legionellales* and others. *E. coli* is responsible for a vast majority of *Escherichia*-related pathogenesis, and other members of this genus have also been implicated in human diseases [87,88]. *Salmonella* species are known intracellular pathogens and certain serotypes are responsible for illness [89]. Altogether these findings suggest that altered structure of intestinal microbiota is related to the pathogenesis of diseases. 

ABs can affect the colonization resistance of host. Treatment with cefoperazone [90], tigecycline [79], clindamycin [80], or clindamycin in combination with a five-antibiotic cocktail in C57BL/6 mice had decreased the colonization resistance, as a result of a decrease in *Lachnospiraceae* and *Barnesiella* and an increase in *Lactobacillaceae* and *Enterobacteriaceae.* These results were largely consistent with human studies [91,92].

Effect of ABs on GM can be persistent. Fouhy et al. (2012) [84] evaluated the short-term recovery of the GM following parenteral ampicillin and gentamicin treatment for infant within 48 hours of birth. It was shown that the abundance of *Proteobacteria* remained significantly higher and the number of different *Bifidobacterium* species was reduced in the infants after 8 weeks of treatment with ABs. It is, thus, obvious that the use of certain ABs in early life can significantly affect the evolution of the infant GM. Another study investigated the short and long-term effects of macrolides on 2–7 year old children (*N* = 142), and found depletion of *Actinobacteria*, increased abundance of *Bacteroidetes* and *Proteobacteria* and increased macrolide resistance, which can persist for over 6 months. Additionally, it was mentioned that the use of macrolides in early life increased the risk of asthma and weight gain [85]. A study in mice reported that *Bacteroidetes* was drastically reduced following treatment with the antibiotic mixture of ampicillin, streptomycin, and clindamycin and never fully recovered after cessation of ABs treatment [81].

The literature regarding the role of altered GM in the development of ABs-related side effects, however, is scarce. The current understanding is that oral intake of ABs lead to disturbance of composition and more importantly the metabolism of GM, which might correlate with disrupted physiology of the host. Study in mice treated with combinative ABs of penicillin, vancomycin and chlortetracycline revealed significant alterations of microbial structure, and altered regulation of hepatic metabolism of lipids and cholesterol, as well as increase of the copies of key genes involved in the metabolism of SCFAs synthesis in fecal and cecal samples [93]. Metagenomic analysis in mice receiving early-life therapeutic-dose pulsed tylosin showed that tylosin intervention decreased the modules involved in glycolysis, gluconeogenesis and tRNA biosynthesis and increased the modules involved in citric acid cycle and nucleoside and amino acid biosynthesis [94]. A study in piglets treated with a mixture of ampicillin, gentamicin and metronidazole also indicated that altered GM was associated with increased metabolism of aromatic amino acids and decreased expression of neurotransmitter in hypothalamus [95].

## 3. Probiotics as a Potential Tool in Contaminants Remediation

Increasing evidence demonstrated that oral supplementation of probiotics is one of the effective strategies for protection against foodborne contaminants-induced toxicity. In general, probiotics applied in toxicant remediation are selected based upon their safety and viability during passage through the GIT [97] and importantly their capacity of contaminants adsorption [98]. The probiotic effects on the hosts are usually assessed by monitoring the individual growth, measuring the amount of pollutant and related biomarkers in tissues, and analyzing the compositional and functional changes of stool microbiota using 16S rRNA sequencing in murine and other models. Nonetheless, the interaction between probiotics and the GM is still poorly understood.

### 3.1. Role of Probiotics in HMs Remediation In Vivo

The protective effects of probiotics against HMs toxicity have been extensively studied. Supplementation of single probiotic or a combination of probiotics in mammalians has shown positive results in alleviating the toxicity of HMs including Cd, Hg, Cr, As and Pb (Table 2). 

**Figure 1:**
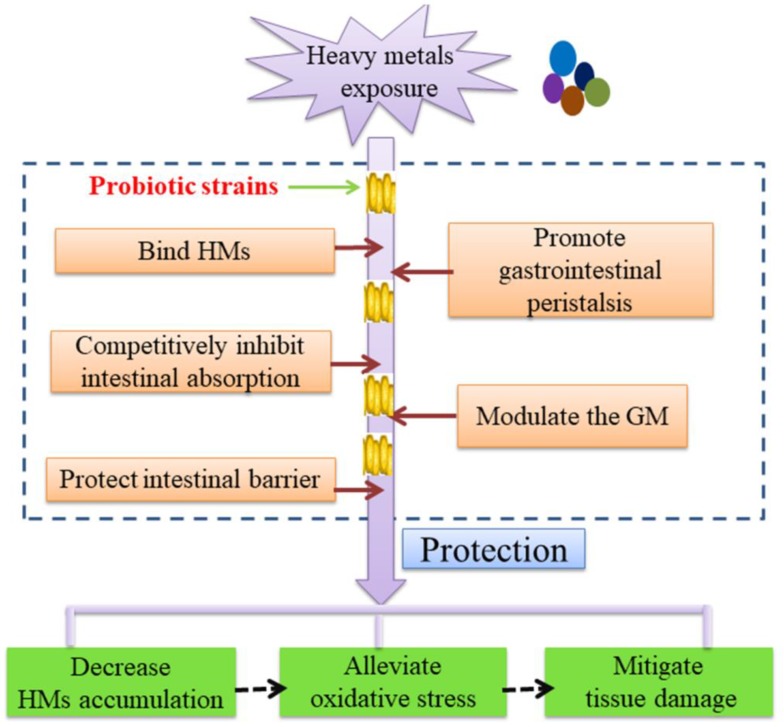
Proposed protective mechanisms of probiotics on HMs remediation in vivo.

Probiotics utilized to reduce the toxicity of HMs are generally *Lactobacilli*, as they have excellent binding capacity for HMs, evidently lowering the availability of HMs for the host [99]. It has also been speculated that living probiotic strain *L. plantarum* CCFM8610 might competitively inhibit the intestinal absorption of Cd by increasing the dissolution and uptake of divalent essential elements like Ca, Mg, and Fe [18]. Probiotic strains can also promote gastrointestinal peristalsis, hence the excretion of HMs in feces is facilitated [18]. Furthermore, probiotic strains can limit the entrance of HMs by enhancing intestinal barrier function and regulating tight junction of epithelium of small intestine. Administration of *L. plantarum* CCFM8610 reversed all of the reductions of mRNA expression of tight-junction proteins (ZO-1, ZO-2, occludin, and claudin-1) caused by Cd exposure, decreased intestinal permeability and reduced Cd leakage into systemic circulation [50]. Preventing systemic absorption of HMs by probiotics thus leads to alleviation of oxidative stress in various tissues and consequent mitigation of tissue damages as reported [100,101,102]. For example, co-treatment of *L. plantarum* CCFM8610 and Cd cause a decreased production of metallothionein and downregulation expression of genes in the mitogen-activated protein kinases (MAPK) pathways in the liver [19]. Metallothionein has a high affinity for divalent cations [103] and the MAPK pathway is associated with reactive oxygen species production [19]. More recently, evidences have suggested that probiotics play a role in restoring the altered composition and function of GM induced by HMs. *L. reuteri* DSM17938 intervention contributed to restoring intestinal homeostasis in patient with low-Ni diets and the increase of lactic acid bacteria (LAB) biodiversity [104]. In the case of reducing Cr (VI) toxicity in mice, the crucial role of probiotic strain *L. plantarum* TW1-1 in maintaining GM homeostasis and enhancing Cr (VI)-reduction ability of intestinal bacteria was underscored [29]. The proposed protective mechanisms of probiotics on HMs remediation are shown in Figure 1.

To date, almost all studies on the efficacy of probiotics were carried out in animals, the only case reported in human was that of *L. rhamnosus* GR-1 (LGR-1)-supplemented yogurt which protected against the absorption of As and Hg in pregnant women and children [105]. Moreover, the effect of HM bioremediation by probiotics is strain-dependent and specific. Although the strain LGR-1 was effective in reducing Hg and As absorption, it could not significantly reduce the blood levels of Pb and Cd in populations, indicating the need for specific probiotics or cocktails of probiotics for protection against different types of HMs.

### 3.2. Probiotics’ Role in Pesticides Remediation In Vivo

Expensive drugs have been developed and long-time therapies have been employed to fight against damages caused by pesticides [106]. More economic alternatives are hence needed to reduce the adverse effects of pesticides. Mounting evidences have highlighted probiotics in mitigating the adverse effects of pesticides (Table 2), and their protective mechanisms are summarized as below. First, *Lactobacilli* protect against pesticides-induced oxidative stress and downstream cellular damage. A few researches have shown that supplementation with *L. plantarum* BJ0021 can decrease oxidative stress and MDA concentration in liver and kidney induced by endosulfan [107]. Another study showed *L. casei* ATCC334 could decrease DNA damage in rats exposed to a carcinogen 1, 2-dimethylhydrazine [54]. Second, probiotics maintain the integrity of intestinal barrier and reduce the absorption of pesticides [108]. It was found that *L. plantarum* MB452 enhanced the expression of tight junction proteins occludin, ZO-1, ZO-2, and cingulin in the Caco-2 intestinal cell-line [109]. Probiotics *L. rhamnosus* strain GG (LGG) and LGR-1 reduced the absorption of parathion or CP in a Caco-2 Transwell model [21]. Third, recent studies found that a few probiotics, mainly *Lactobacillus* from dairy products and wheat, were capable of degrading OCP enzymatically with phosphohydrolase [98,110]. Fourth, *Lactobacilli* stimulate host’s own immunity and detoxification mechanisms to resist pesticides and pathogen invasion. In the study using pattern insects, *L. casei* was found stimulating phase-II detoxification system and rescued malathion-induced physiological impairments in *Caenorhabditis elegans* [20]. Probiotic *L. plantarum* ATCC14917 has shown to stimulate immunity, and lower the pathogenic microorganism (*Serratia marcescens*) infections in fruit flies exposed to imidacloprid [111].

### 3.3. Probiotic Intervention in AAD Patients and Animal Models 

There are a significant number of studies demonstrating the benefits of probiotics in reducing the occurrence of AAD, allergy, lactose intolerance, reduction of cholesterol etc. [112,113]. Patients receiving ABs for treatments are prone to suffer from gastrointestinal disturbances result from damage of the GI mucosal cells and disruption of the gut ecological balance. Probiotics replenish the natural GIT with nonpathogenic bacteria, and are considered as living drugs that help with ABs-associated diseases, without affecting the efficacy of ABs. 

There are many favorable outcomes of probiotics in reducing the risk of AAD in adults and children based on extensive meta-analyses, and only a few studies using probiotics in patients undergoing antibiotic failed to acquire significant effect [114,115]. In a trial study with 246 children, co-treatment of *Saccharomyces boulardii* and ABs has been reported to lower the risk of diarrhea from 20.9% to 8.8% [116]. The updated results of meta-analysis, based on 10 RCTs, also showed that *S. boulardii* effectively prevented AAD in patients, with decrease of risk from 17.4% to 8.2% in adults [117]. The efficacy of LGG for preventing AAD in children and adults has also been evaluated. Treatment with LGG reduced the risk of AAD in patients receiving ABs from 22.4% to 12.3% [118]. The hospital patients who were administered with bio- yogurt containing a combined dose of *L. acidophilus, L. delbrueckii,* subspecies *bulgaricus*, and *S. thermophiles* showed reduced risk from 24% to 12% of AAD [119]. Furthermore, the effects of single and combinative probiotics on preventing AAD were compared. A meta-analysis in 2006 has reported that most of the RCTs used combinations of *Lactobacillus* species which were effective against diarrhea and the relative risk of AAD is 0.43, but there is no high-quality evidence for a single probiotic strain except for *S. boulardii* [120]. 

**Figure 2:**
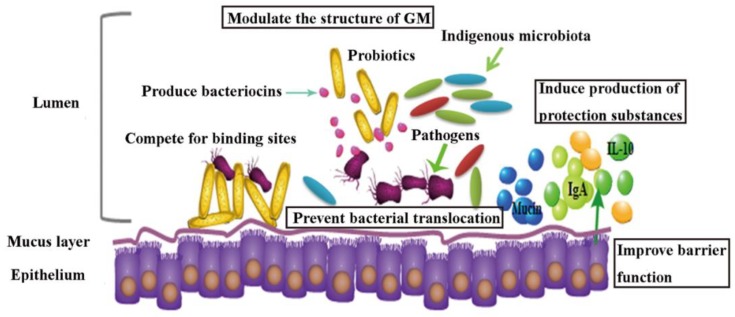
Schematic representation of proposed mechanisms of probiotic action on antibiotics associated diarrhea (AAD).

The efficacy of probiotic strains in reducing the risks of AAD in humans have been evidenced, however, the underlying mechanism of these probiotic strains is less well understood. By reviewing recent literatures (Table 2), probiotics have been proposed to be effective in alleviating ABs-associated diseases through multiple routes (Figure 2): (1) mediating the structure of gut microbial community [81,121,122] by promoting beneficial bacteria and suppressing opportunistic pathogens. A cocktail of *L. rhamnosus A 191, L. acidophilus, B. breve* and *B. longum* significantly caused suppression of gut opportunistic pathogens *Enterobacteriaceae* and promotion of *Firmicutes* following ABs treatment in mice [81]. In another study, it was confirmed that probiotic cocktail of four *Lactobacillus* species JUP-Y4 treatment decreased the levels of *Desulfovibrionales*, and promoted the levels of *Akkermansis* [122]. High abundance of *Desulfovibrionales* were related with Crohn’s disease [123] and human infections [124,125], and *Akkermansis* are biomarkers of intestinal health [126] and inversely linked with the severity of Crohn’s disease and ulcerative colitis [127]. Two probiotics *Phaeobacter inhibens* S4Sm and *Bacillus pumilus* RI06-95Sm in black molly, have been shown to colonize in intestine and reverse mortality caused by streptomycin by inhibiting *Vibrio anguillarum* [121], which are known opportunistic pathogens in fish [128] and are thought to be “r-strategists” capable of rapid growth and virulence in disturbed microbial communities [129,130]. (2) Improving immune function of host by enhancing anti-inflammation [131,132,133,134]. Shi et al. (2017) used two *Lactobacillus* cocktails (LacA and LacB, each contains four strains) to restore the cefixime-induced GM disturbance in mice, and alleviate intestinal inflammation possibly due to beneficial SCFAs production [134]. A probiotic compound of *Streptococcus thermophiles*, *B. breve*, *B. longum*, etc., also reportedly restored the expression of anti-inflammatory cytokine IL-10 completely without affecting pro-inflammatory mediators in mice following broad-spectrum antibiotic treatment. At the meantime, adaptive immunity was also restored, with increase of CD4+, CD8+, and B220+ cell numbers in the intestinal lamina propria [132]. Separate studies demonstrated that *S. boulardii* can up-regulate antitoxin A secretory IgA expression in animal models of AAD [135,136]. (3) Enhancing intestinal barrier function. A probiotic cocktail JUP-Y4 modulated ampicillin induced gut barrier dysfunction and GM disturbance in mice. Increased expression of intestinal epithelial tight-junction proteins, and reduced inflammatory cytokines in the ileum and the colon following JUP-Y4 use contributed to caecum tumefaction attenuation and a decrease in gut permeability [122]. Probiotics have also been shown to increase epithelium mucins production, which is a critical element of the epithelium barrier [137,138]. Probiotics also assist in producing antagonistic activity like bacteriocins against pathogenic bacteria, and inhibiting bacterial translocation by competing for receptors or adhesion to endothelial cells [139,140,141].

## 4. Conclusions and Future Perspectives

The foodborne contaminants, such as HMs, pesticides and ABs, cause harmful effects on animal and human health. GM is a major player in the remediation of these contaminants. Both contaminants-induced toxicity and impaired structure and metabolic activity of GM have significant impacts on target organs, causing tissue damage and other disease. Dietary supplementation with probiotics appears to be a promising adjunct intervention for effectively reducing the damage caused by foodborne contaminants and re-balancing the GM of humans and animals under a constant threat of pollutants. 

The understanding of host-GM interactions must be further developed using a series of techniques such as metagenomics, metatranscriptomics and metabonomics, to provide meaningful insights into the mechanisms of GM, and to clarify the causal relationship between GM and GM-associated symptoms. Additionally, this work needs to be extended to human studies, as majority of research on contaminants remediation using probiotics comes from animal models, rather than humans. Meanwhile, almost all of current studies on the GM and contaminants solely rely on stool microbiota, which is part of the GM and may yield limited conclusions [146]. Hence gut mucosal sampling should also be considered in future studies. Furthermore, the colonization of probiotics in human may vary from person to person, depending on factors such as the composition of individual community, the composition of the colonizers, and intrinsic host factor [147]. Thus, in future applications with probiotics in human, personalized probiotic regimen based on the consumer at different contexts must be considered.

## Figures and Tables

**Table 1:** Recent studies on the effects of foodborne contaminants on hosts and GM.

Type	References	Models	Pollutants and Dosage	Outcomes	Main Conclusion on GM
HMs	[30]	Mice	Cd at 10 mg/L for 10 weeks	Hepatic inflammation, energy metabolism dysregulation	*Firmicutes*↓, *Bacteroidetes*↑, γ- *Proteobacteria*↓
	[41]	Mice	Pb at 32 ppm for 2 weeks	Bodyweight ↑	*Firmicutes*/*Bacteroidetes*↑, *Desulfovibrionaceae*↑, *Barnesiella*↑, *Clostridium XIVb*↑, *Lactococcus*↓, *Enterorhabdus*↓, *Caulobacterales*↓
	[40]	Mice	As at 10 ppm for 4 weeks	Perturbed lipid metabolites, indole-containing metabolites, isoflavone metabolites, and bile acid metabolites	*Firmicutes*↓, *Bacteroidetes*↑
	[29]	Mice	Cr (VI) at 2 mM for 7 weeks	Oxidative stress↑, liver damage, GM disturbance	*Bacteroidetes*↑, *Tenericutes*↑, *Firmicutes*↓, *Paraprevotellaceae*↑, S24-7↑, *Lachnospiraceae*↓
Pesticides	[58,60]	Rats	Chlorpyrifos at 0.3 or 3.0 mg/kg bodyweight/day for 9 weeks	Obese and diabetic phenotypes↑, bacterial translocation↑	*Sutterella*↑, *Candidatus arthromitus*↓, *Olsenella*↑ *Clostridium sensu stricto* 1↑, *Amphibacillus*↑, *Enterorhabdus*↑, *Alloprevotella*↑
	[65]	Mice	Malathion at 2 mg/L in drinking water (∼0.6 mg/kg bodyweight/ day) for 13 weeks	Motility and pathogenicity↑	*Corynebacterium*↑, S24-7↑, *Planococcaceae*↓, *Christenseneellaceae*↓, *Clostridium*↑, *Lachnospiraceae*_Other↓, *Anaerostipes*↓, *Blautia*↓, *Dorea*↓, *Roseburia*↓, *Mogibacteriaceae*↑, *Akkermansia*↓,
	[66]	Mice	Diazinon at 4 mg/L for 13 weeks	Taurine level↑, glycine acetyltransferase and threonine dehydrogenase↓ in male mice	*Bacteroidaceae_Bacteroides*↑, *Burkholderiales*_Other↑, *Clostridiaceae*_Other↑, *Erysipelotrichaceae_Coprobacillus*↑, *Lachnospiraceae_Butyrivibrio*↓, *Lachnospiraceae_Shuttleworthia*↓, *Staphylococcaceae_Staphylococcus* ↓
	[68]	Mice	p,p’-dichlorodiphenyldichloroethylene and β-hexachlorocyclohexane at 1 and 10 mg/kg body weight/day, for 8 weeks, respectively	Bile acid reabsorption in the terminal ileum and compensatory↓, bile acid and hydrophobicity↑, the genes expression on synthesis of bile acids in the liver↑	*Firmicutes*↑, *Proteobacteria*↑, *Bacteroidetes*↓, *Verrucomicrobia*↓, *Actinobacteria*↓
	[70]	Gold Fish	Pentachlorophenol at 0, 10, 50, and 100 μg/L for 28 days	Body weight and liver weight↓, oxidative stress↑, liver damage↑	*Bacteroidetes*↑, *Firmicutes*↓, *Bacteroides*↑, *Chryseobacterium*↓, *Microbacterium*↓, *Arthrobacter*↓, *Legionella*↓
ABs	[72]	Zebrafish	Imazalil at 100 and 1000 μg/L for 1, 7 and 21 days	Glucokinase↓, hexokinase 1↓, pyruvate kinase↓, cytosolic Phosphoenol pyruvate carboxykinase (Pepckc) in liver ↓	*Bacteroidetes*↓, *Firmicutes*↑
	[71]	Mice	Imazalil at 25, 50 or 100 mg/kg body weight daily for 4 weeks	Genes related to glycolysis and lipid metabolism↓	*Lactobacillus*↓, *Bifidobacterium*↓ *Deltaproteobacteria*↑, *Desulfovibrio*↑
	[96]	Rats	Epoxiconazole at 4 and 100 mg/kg body weight/day for 90 days	Weight of the liver and kidney↑, total bilirubin and cholinesterase in serum↓, blood glucose↑	*Firmicutes*↓, *Bacteroidetes*↑, *Proteobacteria*↑, *Lactobacillaceae*↓, *Bacteroidaceae*↑, *Enterobacteriaceae*↑, *Lachnospiraceae*↑
	[81]	Mice	The mixture of ampicillin, streptomycin, and clindamycin at 1 mg/mL for 2–4 week	The ceca size↑, a deeper shade of brown in ceca	Microbial diversity↓, *Bacteroidetes*↓, *Stenotrophomonas*↑, *Xanthamonas*↑
	[95]	Piglets	The mixture of ampicillin, gentamicin, and metronidazole at 150, 4, and 30 mg/kg/day, respectively, for 25 days	Neurotransmitters in blood and hypothalamus↓, amino acids in feces, blood and hypothalamus↓	Microbial diversity in feces↓, *Firmicutes*↑, *Actinobacteria*↑, *Streptococcus*↑, *Lactobacillus*↑, *Bifidobacterium*↑, *Blautia*↑, *Klebsiella*↑, *Euryarchaeota*↓, *Spirochaetes*↓, *Tenericutes*↓, *Ruminococcus*↓, *Clostridium*↓, unclassified *Clostridiales*↓, *Christensenella*↓, *Methanobrevibacter*↓, *Prevotella*↓

↑: Increase of relative abundance of the species or the severity of the outcomes; ↓: Decrease of relative abundance of the species or the severity of the outcomes.

**Table 2:** Recent studies on the protective effects of probiotics against foodborne contaminants toxicity.

Type	References	Models	Contaminants Dosage	Supplementation Dosage	Main Conclusion
HMs	[142,143]	Rats	Cd	CdCl_2_ at 70 ppm, the mixture of *L. acidophilus Rosell*-52, *L. rhamnosus Rosell*-11 and *B. longum Rosell*-175 (5 × 10^8^ CFU/g food) for 5 weeks	Marked decrease genotoxicity and the toxicity to lactobacilli, promoted Cd excretion in feces; decreased Cd in body; relieved liver and kidney damage, increased the number of *L. acidophilus* in feces
	[144]	Rats	Hg	A total of 0.5 mL HgCl_2_ at 20 μg/mL and 1ml *B. coagulans* and *L. plantarum* CNR273 (10^9^ CFU/mL) daily for 48 days	Marked increase Hg excretion in feces; reduce Hg levels in liver and kidney; prevent oxidative stress; reduce liver and kidney damage; increase the number of fecal LAB and the total bacteria counts
	[145]	Mice	Pb	A total of 2 mg (CH_3_COO)_2_Pb·3H_2_O in 0.4 mL plain water, *L. bulgaricus* KLDS1.0207 1 × 10^10^ (high dose), 1 × 10^9^ (medial dose) and 1 × 10^8^ (low dose) CFU/mL in 0.4 mL skim milk	Lower mortality rates, increased Pb excretion in feces, decreased tissue Pb enrichment, improved the antioxidant in the liver and kidney, and relieved renal pathological damage
	[101]	Rats	As	NaAsO_2_ at 1.0 mg/100 g body weight, the mixture of *L. acidophilus*, *L. rhamnosus*, *B. longum*, and *S. boulardii* at 0.25 mg/100 g body weight for 16 days	Reduction of oxidative stress, inflammation in uterine, protection against mutagenic uterine DNA-breakage, necrosis, ovarian-uterine tissue damages
	[29]	Mice	Cr (VI)	A total of 1mM K_2_Cr_2_O_7_ in drinking water, *L. plantarum* TW1-1 (1 × 10^9^ CFU/once every other day) for 7 weeks	Promoted Cr excretion in feces, reduced Cr accumulation in tissues; decreased oxidative stress and damage in liver; partially restored the GM community
Pesticides	[107]	Rats	Endosulfan	Endosulfan at 4 mg/kg bodyweight from the 6th to 20th day of gestation, *L. plantarum* BJ0021 0.1 mL per os and one hour before the administration endosulfan	Significantly reduced the cholesterol level and marked depletion of hepatic enzymes, decreased the number of apoptotic nuclei in kidney
	[20]	*Caenorhabditis elegans*	Malathion	Exposure to malathion at 300 mM for 4 h at 20 °C after administration *L. casei* liquid cultures of 0.1 OD at 600 nm for 4 h	Reproduction protection with increase of rate of egg laying and brood size, and rescued locomotion of *C. elegans*
	[21]	*Drosophila melanogaster*	Chloropyrifos parathion	Co-exposure 10 μM chloropyrifos parathion and 100 μL *L. rhamnosus* GG (10^9^ CFU) for 12 days	Prolonged overall survival and decreased early deaths
ABs	[81]	Mice	Different ABs	Ampicillin, Streptomycin, and Clindamycin at 1 mg/mL, A cocktails of *L. rhamnosus* A191, *L. acidophilus*, *B. breve*, *B. longum* (4 × 10^9^/mL) at 0.1 mL/mouse for 2 weeks	Lead a rise in microbial diversity; small increase in *Firmicutes*, increase in *Enterobacteriaceae*, and a bloom of *Anaerotruncus*, decrease in *Xanthamonas*
	[121]	Fish	Streptomycin sulfate	A total of 200 g/mL of streptomycin sulfate daily for 13 days, 1 × 10^5^ CFU/mL *P. inhibens* S4Sm and *B. pumilus* RI06-95Sm daily for 5 days following ABs treatment	Probiotics can colonize fish microbiome, decrease mortality in fish with subtle GM changes
	[122]	Mice	Ampicillin	Ampicillin (500 mg/kg) twice-daily for 14 days, a cocktail of *L. plantarum*, *L. casei*, *L. rhamnosus* and *L. helveticus* (2 × 10^9^ CFU/0.2 mL/dose) for 4 weeks	Restore diversity of GM, decrease *Firmicutes*, reduce *Desulfovibrionales*, *Dorea*, *Ruminococcus*, *Clostridia* and *Helicobacter*, enrich *Akkermansia*, *Alistipes* and *Porphyromonadaceae*
